# Natural Polyhydroxy Flavonoids, Curcuminoids, and Synthetic Curcumin Analogs as α7 nAChRs Positive Allosteric Modulators

**DOI:** 10.3390/ijms22020973

**Published:** 2021-01-19

**Authors:** Marta Ximenis, José Mulet, Salvador Sala, Francisco Sala, Manuel Criado, Rosario González-Muñiz, María Jesús Pérez de Vega

**Affiliations:** 1 Instituto de Química Médica-Consejo Superior de Investigaciones Científicas (IQM-CSIC), Juan de la Cierva 3, 28006 Madrid, Spain; ximenis@edu.k.u-tokyo.ac.jp (M.X.); iqmg313@iqm.csic.es (R.G.-M.); 2 Instituto de Neurociencias, Universidad Miguel Hernández-CSIC, 03050 Sant Joan d’Alacant, Spain; jmulet@umh.es (J.M.); salvador.sala@umh.es (S.S.); fsala@umh.es (F.S.); manuel.criado@umh.es (M.C.)

**Keywords:** curcuminoids, tetrahydrocurcuminoids, α7 nicotinic receptors, positive allosteric modulation

## Abstract

The α7 nicotinic acetylcholine receptor (α7 nAChR) is a ligand-gated ion channel that is involved in cognition disorders, schizophrenia, pain, and inflammation. Allosteric modulation of this receptor might be advantageous to reduce the toxicity in comparison with full agonists. Our previous results obtained with some hydroxy-chalcones, which were identified as positive allosteric modulators (PAMs) of α7 nAChR, prompted us to evaluate the potential of some structurally related naturally occurring flavonoids and curcuminoids and some synthetic curcumin analogues, with the aim of identifying new allosteric modulators of the α7 nAChR. Biological evaluation showed that phloretin, demethoxycurcumin, and bis-demethoxicurcuming behave as PAMs of α7 nAChR. In addition, some new curcumin derivatives were able to enhance the signal evoked by ACh; the activity values found for the tetrahydrocurcuminoid analog **23** were especially promising.

## 1. Introduction

The α7 acetylcholine nicotinic receptors (α7 nAChRs) are excitatory neurotransmitter receptors of the ligand-gated ion channels superfamily. They are involved in neurological/cognition disorders, as well as in pain and inflammation processes [1,2,3], being potential therapeutic targets for the treatment of several diseases such as Alzheimer or schizophrenia [4,5,6,7]. Therefore, the development of new agents that target these receptors has great therapeutic significance. Research efforts until now lead to the identification of some clinical candidates; however, more research will be needed in order to identify new more potent and effective ligands that could end in clinical candidates.

In this context, positive allosteric modulators (PAMs) might be advantageous, since they facilitate receptor responses without directly interacting with the agonist binding site, which can avoid many of the adverse effects. PAMs are compounds that modulate the α7 nAChRs activity, binding to sites different to that of the endogenous ligand. This could represent an advantage regarding selectivity for α7 nAChRs versus other nicotinic receptors and also versus 5-HT_3_ serotonine receptors that bear a high structural homology with the α7 nAChRs, avoiding cross-reactivity and consequently undesired effects [8,9]. According to their capacity to delay the desensitization of the receptors, two kinds of PAMS can be distinguished, Type I and II. Type I PAMs do not practically affect the agonist-induced desensitization, while Type II PAMs affect receptors’ kinetics causing a delay in desensitization and, as a consequence, a prolonged channel opening [10]. More recent studies lead to identify other types of allosteric modulators, like the silent allosteric modulators (SAMs) that block allosteric potentiation without interfering the responses of orthosteric agonist. There are also compounds which can activate the receptor through sites different from the orthosteric sites, not affecting the PAM action and without needing the presence of the orthosteric agonist, the so called ago-PAMs. Lastly, the negative allosteric modulators (NAMs), are able to allosterically block the open channel [11]. The interest for α7 nAChRs PAMs is still alive and is evidenced by the approval of a number of activators to be advanced into clinical trials [12,13], for instance, AVL-3288, currently in phase I for the treatment of schizophrenia [14,15], or Encenicline (EVP-6124), in phase III for smoke cessation [16,17].

In the search for new chemical entities able to modulate the α7 nAChRs, a few years ago we evaluated a heterogeneous collection of commercially available small-molecule natural products. As a result of this screening, a natural flavonoid with a chalcone structure, isoliquiritigenine (4,2′,4′-trihydroxychalcone), was identified as a selective PAM of the α7 nAChRs. This finding led to the evaluation of a collection of chalcones and related compounds, leading to the discovery of two new families of potent PAMs of the α7 nicotinic channels—polyhydroxychalcones [18] and polyhydroxydiphenyl propanones. This permitted the identification of a new chemotype of α7 nAChRs PAMs and compounds that exhibit promising analgesic and neuroprotective activities [19,20,21].

We report here the extension of the evaluation to a small collection of polyhydroxy flavonoids of natural origin together with known curcuminoids and new synthetic curcumin derivatives in an effort to explore the potential of these scaffolds in our ongoing interest for identifying new PAMs of the α7 nAChR with improved potency and efficacy. The structural similarity of flavones, chalcones, and curcumins invite to think that they could behave as α7 nAChRs PAMs interacting with the receptors in the same way as the previously described chalcones. Preliminary studies of selected compounds in a primary screening led to the identification of some curcuminoids able to significantly enhance the acetylcholine-evoked currents.

## 2. Results and Discussion

### 2.1. Evaluation of Polyhydroxy Natural Products (NPs)

Considering the biological activity reported for several natural products (NPs) in reference to the modulation of ion channels, and the results previously found for our chalcone collection, we decided to investigate the ability of a small collection of commercially available NPs to activate α7 nicotinic channels. The chemical structure of the selected PNs was closely related to that of chalcones, mainly flavonoids and curcuminoids (Table 1). In our first screening of a structurally diverse heterogeneous collection of PNs, we already identified a flavonoid, isoliquiritigenin, as a PAM of the α7 [18]. Isoliquiritigenin, is a polyhydroxy substituted chalcone extracted from the licorice plant that was widely used in Chinese medicine and is described to have a wide spectrum of therapeutic properties, among other neuroprotective effects [22,23]. Flavonoids have been extensively used in traditional herbal medicine [24], having important biological effects associated to a wide spectrum of pharmacological activity. They play preventive and therapeutic roles in several pathological processes like arthritis [25], neurodegenerative [26,27,28] and cardiovascular processes [29], cancer [30], or pain [31,32]. Several studies revealed that the biological actions of some flavonoids are in part mediated by their ability to modulate the activity of some ion channels, for instance, the cholinergic nicotinic channels.

The findings reported above prompted us to comparatively screen a small, more directed collection of polyhydroxy-substituted naturally occurring and commercially available compounds with a chemical structure closely related to the chalcones previously identified by our group as PAMs of α7 nAChRs (Table 1). The selected compounds included some flavonoids, compounds **1** to **10**, a diphenyl-2-propanone, phloretin **11**, curcumin **12**, and the curcuminoids **13** and **14**. The results of the evaluation in a cellular assay, in *xenopus* oocytes selectively expressing α7 nAChRs, to determine its capacity to enhance the ion currents evoked by acetylcholine is shown in Table 1.

Among the results found, it is worth noting the values exhibited by some flavonoids, like quercetin (**2**), genistein (**4**), kaempferol (**6**), and especially phloretin (**11**). This last compound has a polyhydroxy-substituted diphenyl-2-propanone scaffold, similar to one of our already explored series that led to the identification of new potent PAM derivatives, as mentioned above [19]. Comparatively, some of our diphenyl-2-propanones are better PAMs of α7 nAChRs than phloretin itself. On the other hand, curcumin (**12**), especially the curcuminoids **13** and **14**, significantly enhance the ACh-induced ion currents. The last two compounds, **13** and **14**, showed strong potentiation, being able to increase the currents in more than 4-fold (408 and 469%, respectively).

Previous work described concerning some of the compounds that showed good results in our assay, already revealed their capacity to modulate ion channels and in particular α7 nAChRs. Therefore, in 2011, Dey R. et al. performed a virtual screening study of about 5000 small molecules, at three potential binding sites looking for allosteric modulators of the α7 receptors, which resulted in the identification of genistein (**4**) as one of the 100 compounds that could behave as PAM of the α7 receptors [33]. Moreover, the ability of genistein to potentiate the α7 nAChRs-mediated responses was already reported in 2005 by Robin A. J. and coworkers [34]. Later work published in 2007 pointed to its behavior as PAM [35], which was in accordance with the findings of the virtual screening carried out by Dey R. and Chen L. in 2011 [33]. Genistein promotes the overexpression of α7 nAChRs in adipose tissue of isolated mature adipocytes from obese subjects, where the levels of these protein are down regulated together with an inflammatory profile providing evidence of its role in the modulation of these receptors [36,37]. In agreement with that, work recently reported by Nielsen et al. evidenced that genistein and quercetin (**2**) were able to enhance the α7 nAChRs currents evoked by acetylcholine (ACh) in oocytes expressing α7 nAChRs, showing a PAM profile [38].

On the other hand, curcumin (**12**), a yellow pigment isolated from the rhizome of *curcuma longa* (turmeric), extensively used in traditional medicine for thousands of years, has been widely studied because of its uncountable therapeutic properties [39], from cancer [40,41] and arthritis [42] to neurodegenerative diseases [43] like Parkinson [44] and Alzheimer [45], as well as pain [46]. Its biological activity is mainly attributed to its particular chemical structure, having the advantage of low toxicity. Moreover, its chemical structure is very closely related to that of chalcone, which moved us to consider curcumin for our purposes, together with curcuminoids, demethoxycurcumin (**13**), and bisdemethoxycurcumin (**14**). Additionally, the interaction of curcumin with ion channels was also studied, trying to shed light into the underlying molecular mechanisms, and was broadly reviewed [47,48]. In fact, curcumin itself has recently been reported as an α7 nAChRs PAM, reversing nociception in mouse models [49,50]. Moreover, a recent work that describes the curcumin ability to activate vagal afferent neurons increasing neuron excitability suggests the mediation of the α7 nAChRs [51].

**Figure 1:**
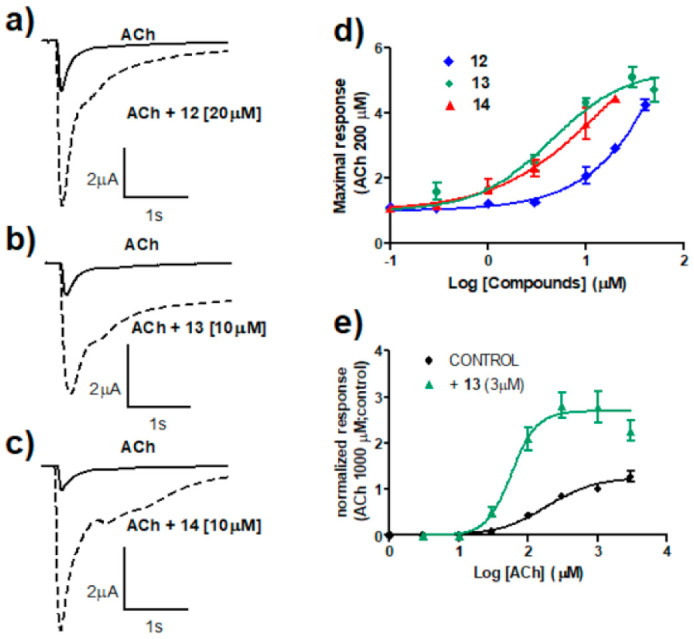
Characterization of curcumin (**12**) and two curcuminoids (compounds **13** and **14**). (**a**–**c**) Ionic currents recorded in representative oocytes expressing human α7 nAChRs. Currents were evoked by 600 ms applications of ACh 200 μM in the absence (continuous line) and in the presence (dotted line) of 20 μM of **12** (panel (**a**)) or 10 μM of **13** (panel (**b**)) and **14** (panel (**c**)). All currents were recorded at a holding potential of –80 mV. (**d**) Concentration–response relationships for the potentiating effect of **12**, **13**, and **14** co-applied with ACh 200 μM. Continuous lines represent the fit to the Hill equation. Limited solubility of these compounds, especially **12** and **14**, hindered the use of higher concentrations in order to adequately complete the curves, so that the following values should be considered with caution. Thus, maximal potentiating effects were estimated as 11 ± 3-, 5.5 ± 0.3-, and 5.5 ± 1.0-fold for compounds **12**, **13**, and **14**, respectively. The EC_50_ values were 85 ± 35, 4.9 ± 1.0, and 6.9 ± 3.1 μM for **12**, **13**, and **14**, respectively. (**e**) Concentration–response curves of peak currents elicited by ACh in control conditions or in the presence of 3 μM **13**. Data have been normalized to the peak current obtained in control conditions with 1 mM ACh. Lines are fit to the Hill equation with parameters (I_max_, EC_50_, nH): control (1.25, 176 μM, 1.25), 13 (2.70, 57 μM, 2.36). Data were taken from 3–7 oocytes from at least 2 donors.

Compounds **12**, **13**, and **14** were characterized in more detail (Figure 1). Figure 1a–c features the potentiating effect that was observed in ACh-evoked ionic currents. In all cases, the currents decayed relatively fast; hence, the changes in kinetics were similar to those found for Type I PAMs. Recently, it has been reported that curcumin acts as a Type II PAM of α7 nACRs. Despite that, in the presence of this compound, α7 currents were observed to decay over time [50]. We and others (see, for instance, Grønlien et al., 2007 [35]) consider that Type II PAMs act by increasing maximum responses but also avoiding the typical desensitization of α7 nAChRs, which is not the case of curcumin. The three compounds potentiated α7 currents in a concentration-dependent manner (Figure 1d). Compound **12** seemed to be the most effective (1100% enhancement of ACh-induced currents, although this result should be considered only approximated), whereas compound **13** seemed to be the most potent (EC_50_ 4.9 µM). Our results regarding the strong potentiating effect of curcumin (compound **12**) confirm previous ones by El Nebrisi et al., although they differ quantitatively [50]. This could be due to the different conditions used in the experiments. For instance, these authors used a 20-fold lower concentration of ACh in their concentration–response experiments. Moreover, pre-treatment time was different, 10 min vs. 2 min in our case. Dose–response curves for ACh in the absence and in the presence of compound **13** were obtained (Figure 1e). Compound **13** induced a slight leftward shift (3-fold) and, most importantly, a 2-fold increase in apparent efficacy.

The strong potentiation of ACh-induced currents found for demethylated curcuminoids, encouraged us to synthesize some new curcumin analogues, taking previous results found for the chalcone and diphenylpropanone series as a model [18,19].

### 2.2. Design and Synthesis of New Curcumin Derivatives

Despite the outstanding and multiple therapeutic properties of curcumin, there is a major limitation that restricts its therapeutic use, its poor pharmacokinetic properties, low aqueous solubility, low metabolic stability, and low oral bioavailability [52]. This promoted important research efforts to improve its therapeutic profile while keeping the beneficial biological properties, like modification of its chemical structure leading to the preparation of different curcuminoids. Among them, dimethoxycurcumin (DiMC), a synthetic analogue with higher metabolic stability [53], and tetrahydrocurcumin (THC), both exhibiting pleiotropic biological activities. Tetrahydrocurcumin, is one of the major metabolites of curcumin, being equally or even more effective than curcumin in a number of targets [54,55,56]. For the selection of the new curcumin derivatives to be synthesized, we designed tetramethoxy curcuminoids, analogues of DiMC, following the pattern substitution that led to the best results on the previously described chalcone series [18]. Besides, providing the good results found for the propen-2-one series [19], the saturated analogs of chalcones, preparation of the corresponding tetrahydrocurcumins, by reduction of the linker double bonds was also contemplated. Finally, assays to prepare the corresponding fully demethylated analogues were performed.

**Scheme 1:**
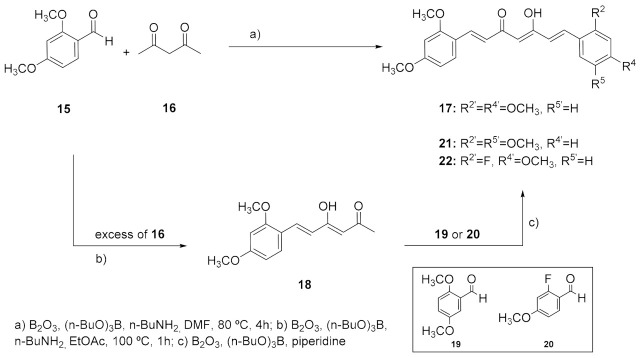
Synthesis of symmetrically substituted (**17**) and asymmetrically substituted (**21**, **22**) curcumin derivatives.

The preparation of the differently substituted curcumin analogues was tackled following the synthetic strategy originally described by Pabon, starting from the appropriate benzaldehydes and 2,4-pentanedione-boric anhydride in EtOAc in the presence of tributyl borate and n-butylamine [57]. The strategy consisted in protecting the highly acidic methylene protons of the 2,4-pentanedione system from the Knoevenagel condensation by forming a boron complex, so that the reaction can only take place at the terminal methyl groups of the diketone [58]. Compound **17**, the tetramethoxy analogue symmetrically substituted in positions 2 and 4 of the phenyl rings, was firstly prepared. However, given the low yield obtained in the synthesis of **17** (17%), an alternative procedure described by Ferrari et al. was tried, that makes use of DMF instead of EtOAc as a solvent (Scheme 1) [59]. This method allowed us to shorten the reaction times as well as to reduce the volume of the solvent. Moreover, the easier reaction workup simplified the isolation and separation of **17** from byproducts. On the whole, this one-pot reaction resulted in a higher yield of 52% after crude recrystallization.

Having in mind the good results obtained with the chalcone analogues, and searching for a possible parallelism, the synthesis of asymmetrically substituted curcumins **21** and **22**, was explored. Compound **21** responds to the pattern substitution that led to the best results in the chalcone series, 2,4,2′,5′ -tetrasubstitution. On the other hand, considering the low stability and low bioavailability of curcumin systems, the incorporation of a fluorine atom in the aromatic ring (**22**) was also considered. The C-F bond is particularly strong and thus resistant to metabolic cleavage, given that fluorine is highly electron-withdrawing and reduces the potential for oxidative metabolism. Furthermore, fluorine substitution normally increases bioavailability [60]. The synthesis of these asymmetric analogues was carried out by preparing, in first instance, compound **18**, which required the use of an excess of pentanedione to avoid the aldol condensation taking place at both terminal ends [61]. Subsequent condensation of isolated **18** with the appropriate aldehyde, **19** or **20**, led to the asymmetrically substituted curcuminoids **21** and **22**, respectively (Scheme 1). Unlike what was observed in the preparation of **17**, in the case of the monophenyl intermediate **18**, the use of EtOAc as solvent (27%) led to a slightly better result compared to DMF (15%). Coupling **18** with aldehyde **19** led to **21** in moderate yield (32%). In this case, the use of n-BuNH_2_ as a catalyst led to complex reaction mixtures, but the reaction proceeded using piperidine instead, which finally gave the desired compound **21 [62]**. Fluorine derivative **22** was obtained by coupling **18** with aldehyde **20** using also piperidine as a catalyst. Unfortunately, several attempts to improve the yield and reduce the reaction time by using microwave irradiation and calcium oxide as a catalyst failed to provide the desired products and only complex mixtures were obtained [63].

**Scheme 2:**
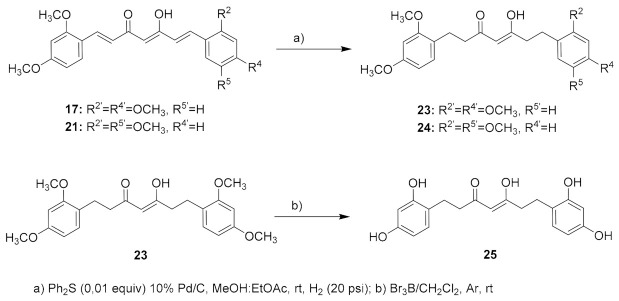
Synthesis of tetrahydrocurcumins **23**-**25**.

Additionally, searching for a parallelism with the chalcone series, the corresponding tetrahydrocurcumin analogues of **17** and **21** were prepared by reduction of the linker-chain double bonds, leading to **23** and **24**. The reduction conditions were similar to those used for the reduction of the chalcones linker, by Pd/C hydrogenation in the presence of diphenyl sulfide, a catalyst poison that permits the selective hydrogenation of the double bonds without affecting the carbonyl groups (Scheme 2) [64].

Finally, preparation of deprotected hydroxyl substituted derivatives of curcuminoid **17** and tetrahydrocurcumins **23** and **24** was attempted by reaction with Br_3_B in CH_2_Cl_2_ under inert atmosphere, as described by McOmie et al. [65]. However, the synthesis showed more difficulties than expected, and only the preparation of analogue **25** was achieved (Scheme 2). In order to determine the most favorable reaction conditions to arrive at the desired totally deprotected analog of **17**, several assays for demethylation (Scheme 2) were performed using different amounts of reagent, 2, 1.5, and 1 equiv, for each reacting group. The best results were found using 1.5 equiv of Br_3_B as shown by the HPLC-MS data of the crude product, although the corresponding tetrahydroxy compound was obtained at quite a low purity (75%, HPLC data). All attempts to purify this material were unsuccessful resulting in decomposition of the product. The reaction worked better for the corresponding tetrahydrocurcumin analogue **23** to give the expected tetrahydroxy compound **25** in a moderate yield (24%), while efforts to isolate the demethylation product derived from the asymmetric curcuminoid **24** failed. A possible explanation could be the rapid oxidation, experimented by the para-dihydroxy substituted ring, which would lead to the corresponding benzoquinone product the presence of which was detected in the MS spectra of the reaction untreatable raw material.

Concerning the structure of the isolated compounds, the formation of the E,E isomer as a unique product was observed in all cases, as shown by the NMR spectra. On the other hand, it is worth noting that curcumins can exist in a tautomeric equilibrium between diketo and keto-enol forms, where the intramolecular hydrogen bond usually predominates in organic solvents, whereas the diketo form was mainly found in aqueous media, being stabilized by an increase in polarity [66] (Figure 1). In view of the NMR spectra, it can be said that the compounds were isolated in the enol form, with the only exception of tetrahydroxy tetrahydrocurcuminoid **25**. In the HPLC chromatogram of this compound only one peak was observed, which indicates a high degree of purity, and that peak corresponds to the expected mass. However, in the NMR spectra, the presence of the two tautomeric forms was in fact detected. This is confirmed by the identification of two signals for C_4_, corresponding to the enol and keto forms, in the ^13^C spectrum and to linker H_4_ in the ^1^H-NMR spectrum. Additionally, while in the ^1^H-NMR the signals of the aliphatic protons were overlapped with that of the solvent, the ^13^C spectra denotes the presence of a duplicated number of signals in the aliphatic region, which were tentatively assigned on the basis of the bidimensional HSQC-NMR spectra.

To discard any possible artefacts due to decomposition of the compounds in solution, the chemical stability of the best compounds **17**, **23**, and **25** was checked in aqueous mixtures of H_2_O/acetonitrile 20/80 for a period of two months. The samples were periodically checked in the same HPLC conditions (gradient, column, and equipment), at different time intervals for a period of two months, observing no alteration on the retention times and purity along the tested period.

### 2.3. Biological Evaluation of New Curcumin Derivatives

The obtained compounds were screened to determine their capacity to modulate the α7 nAChR activity (Table 2). Each compound (10 μg/mL) was co-applied with ACh (200 μM) to oocytes expressing homomeric α7 nAChRs previously incubated with the same concentration of compound. Currents were measured and compared with those induced by ACh alone.

Looking at the data altogether, the most significant result was found for tetrahydrocurcuminoid **23**, with 2,4-dimethoxy symmetrically substituted phenyls. No significant PAM properties were found for the 2,4,2′,5′-asymmetric compounds **21** and **24**, the latter even decreasing the ACh-induced currents, while the fluorine-derivative **22** increases them only slightly. On the other hand, the reduction of the unsaturated linker is convenient, because compound **23**, the tetrahydrocurcuminoid analogue of **17**, enhanced the current at a higher percentage than its curcuminoid precursor. The only isolated tetrahydroxyl analogue, **25**, showed a tendency for increasing the current but at a lower percentage compared to its MeO-substituted precursor **23**. Regarding the latter, it is interesting that just the displacement of a methoxy substituent from position 3 to 4 resulted in the conversion of a potentiator into an antagonist (compound **24**).

In parallel with the chalcone/diphenyl propenone series, it can be ventured, that also in this case compounds with the saturated linker chain were able to enhance the α7 nAChR currents evoked by ACh. However, considering the values found for **23** and **25**, on this occasion, the methoxy substitution was preferred over the hydroxyl analogue in contrast with the results found for chalcones and diphenylpropanones, for which methoxy-substituted derivatives inhibited α7 nACh channels, while hydroxyl compounds behaved as α7 nAChR PAMs, increasing the activity promoted by ACh [18,19]. Regarding the results obtained for curcumin **12** and curcuminoids **13** and **14**, the capacity to potentiate the currents increases with the decrease in the number of MeO- groups, the percentage values being higher in the following order **14** > **13** >> **12** (Table 1). However, compared with the new compounds prepared (**17**, **20**-**25**), compound **23**, a 2,4,2′,4′-tetramethoxy tetrahydrocurcumin analogue, is the compound that showed the best value, comparable to that found for BDMC, **14**, the 4,4′-dihydroxy curcuminoid (Table 2).

**Figure 2:**
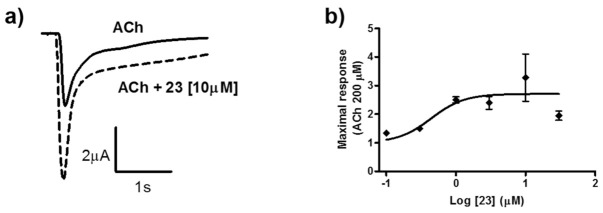
Characterization of compound **23**. (**a**) Ionic currents recorded in representative oocytes expressing human α7 nAChRs. Currents were evoked by 600 ms applications of ACh 200 μM in the absence (continuous line) and in the presence (dotted line) of 10 μM of **23**. All currents were recorded at a holding potential of −80 mV. (**b**) Concentration–response relationship for the potentiating effect of **23** co-applied with ACh 200 μM. Continuous line represents the fit to the Hill equation. Thus, maximal potentiating effect was estimated as 2.7 ± 0.3-fold, and the EC_50_ value was 0.44 ± 0.28 μM. Data are taken from 3–7 oocytes from at least 2 donors.

Compound **23** showed the highest increase in ACh-induced currents (Table 2) and, therefore, was further characterized (Figure 2). As it was previously observed for curcumin and derivatives (Figure 1a), currents potentiated by compound **23** decayed relatively fast (Figure 2a), as in control. Maximal activation of about 3-fold over the control was already observed at a relatively low concentration of 1 μM (see Figure 2b). Compound **23** showed a one and two orders of magnitude higher PAM potency than curcuminoids **13**/**14** and curcumin **12**, respectively.

## 3. Materials and Methods

### 3.1. Chemistry

#### General Methods

All reagents were of commercial quality. Solvents were dried and purified by standard methods. Analytical thin-layer chromatography (TLC) was performed on aluminum sheets coated with a 0.2 mm layer of silica gel 60 F254. Silica gel 60 (230–400 mesh) was used for flash chromatography. Some compounds were purified by flash chromatography on an ISOLERA ONE (Biotage) apparatus using normal phase: SNAP 10 g or 25 g KP-SIL (Biotage® KP-Sil Flash) cartridges and mixtures of ethyl acetate (solvent A) and n-hexane (solvent B) were used as mobile phase. The gradient will be indicated in each case. HPLC-MS was conducted in a Waters 2695 separation module using a Sunfire C_18_ (2.1 × 50 mm, 3.5 μm) reversed-phase column, gradient of 0.1% formic acid in CH_3_CN (solvent A) and 0.1% formic acid in H_2_O (solvent B) as mobile phase, coupled to a Waters 2996 Photodiode Array detector and a Waters micromass ZQ (ESI^+^). ^1^H and ^13^C-NMR spectra were registered in a Varian 300, 400, or 500 MHz spectrometer, using TMS as the internal standard.

Synthesis of Compounds 17, 18, 21 and 22

**(1E,4Z,6E)-1,7-Bis(2,4-dimethoxyphenyl)-5-hydroxyhepta-1,4,6-trien-3-one (17).** A suspension of B_2_O_3_ (4.87 mmol) and 2,4-pentanedione (4.87 mmol) in DMF (6 mL) was stirred for 0.5 h at 80 °C, and then tributylborate (19.48 mmol) was added. After 0.5 h, 2,4-dimethoxybenzaldehyde (8.76 mmol) was added, followed by slow addition of n-butylamine (1.95 mmol in 2 mL of DMF). After stirring at 80 °C for 4 h, the solution was acidified with 0.5N HCl (16 mL) and cooled down to room temperature. The resulting solid was suspended in ice water, filtered, and dried under vacuum. The crude residue was recrystallized from MeOH. Brown yellow prisms (52%). m.p.: 140–142 °C (MeOH) (Lit.[61]: 135–137 °C). HPLC-MS: *t*_R_ = 9.50 (10 min gradient of 40 to 95% of A in B). ^1^H-NMR: (300 MHz, CDCl_3_) δ (ppm): 3.85 (s, 6H, OCH_3_), 3.88 (s, 6H, OCH_3_), 5.80 (s, 1H, H_4_), 6.46 (d, *J* = 2.4 Hz, 2H, 3-H, 3′-H), 6.52 (dd, *J* = 8.5 and 2.4 Hz, 2H, 5-H, 5′-H), 6.63 (d, *J* = 16,0 Hz, 2H, H_2_, H_6_), 7.49 (d, *J* = 8.5 Hz, 2H, 6-H, 6′-H), 7.89 (d, *J* = 16.0 Hz, 2H, H_1_, H_7_), 16.19 (br s, 1H, OH). ^13^C-NMR: (125 MHz, CDCl_3_) δ (ppm): 55.63 and 55.65 (4OCH_3_), 98.6 (C3, 3′), 101.3 (C_4_), 105.5 (C5, 5′), 117.5 (C_5_), 122.6 (C_2_, C_6_), 130.2 (C6, 6′), 135.6 (C_1_, C_7_), 160.0, 162.7, 182.25 (C_3_-OH), 184.0 (CO). MS (ESI+): *m*/*z* 397 [M + H]^+^. HRMS (ESI+): *m*/*z* [M ]^+^ calc for C_23_H_24_O_6_ [M ]^+^ 396.1573, found 396.1581.

**(3Z,5E)-6-(2,4-Dimethoxyphenyl)-4-hydroxyhexa-3,5-dien-2-one (18).** To a solution of 2,4-pentanedione (9 mmol) in EtOAc (5 mL) B_2_O_3_ (2.1 mmol) was added. The solution was stirred at 70 °C for 0.5 h, and then the corresponding aldehyde (3 mmol) and tributyl borate (3 mmol) were added. The mixture was stirred for 30 min, and then a solution of n-butylamine (3 mmol) in EtOAc (5 mL) was added at 85 °C, dropwise over 15 min. The stirring continued for 1 h at 100 °C. The mixture was then hydrolyzed by adding 1N HCl (12 mL) at 50 °C and then stirred at the same temperature for 0.5 h. After that, the organic layer was separated, and the aqueous layer was extracted with EtOAc. The combined organic extracts were washed with water until neutral pH, and finally with brine and dried over anhydrous MgSO_4_. After removal of the solvent under reduced pressure, the crude product was purified by flash HPLC and crystallized from MeOH giving **18** a**s** yellow needles (27%). m.p.: 108–111 °C (MeOH) (Lit.[61] 92–93 °C). Purified by flash chromatography (gradient: 0 to 30 of EtOAc in hexane). HPLC-MS: *t*_R_ = 6.62 (10 min gradient of 30 to 95% of A in B; column Sunfire 4.6 × 15 mm). ^1^H-NMR: (300 MHz, CDCl_3_) δ (ppm): 2.14 (s, 3H, H_1_), 3.84 (s, 3H, OCH_3_), 3.87 (s, 3H, OCH_3_), 5.62 (s, 1H, H_3_), 6.45 (d, *J* = 2.5 Hz, 1H, 3-H), 6.49 (d, *J* = 16.0 Hz, 1H, H_5_), 6.50 (dd, *J* = 8.5 and 2.5 Hz, 1H, 5-H), 7.45 (d, *J* = 8.5 Hz, 1H, 6-H), 7.84 (d, *J* = 16.0 Hz, 1H, H_6_), 15.57 (br s, 1H, OH). MS (ESI+): *m*/*z* 249 [M + H]^+^.

**(1E, 4Z, 6E)-1-(2,4-Dimethoxyphenyl)-7-(2,5-dimethoxyphenyl)-5-hydroxyhepta-1,4,6-trien-3-one (21).** The 2,5-dimethoxy benzaldehyde (**21**) (0.171 g, 1.03 mmol) and B_2_O_3_ (90 mg, 1.29 mmol) was dissolved in EtOAc (2 mL) at 80 °C. To this mixture an EtOAc solution (3 mL) of **18** (159 mg, 0.64 mmol) and (*n*-BuO)_3_B (0.35 mL, 1.40 mmol) was added. After stirring for 0.5 h, the reaction mixture was treated with piperidine (26 μL, 0.26 mmol) at 80 °C for 0.5 h. Then 1N HCl (10 mL) was added at room temperature, and the reaction mixture was stirred overnight. The aqueous phase was extracted with EtOAc, washed with water, and then dried over MgSO_4_. Normal-phase flash chromatography (gradient: 0–30% of EtOAc in hexane) and further crystallization from MeOH led to the desired product **23**. Yellow prisms (32%). m.p.: 115–118 °C (MeOH). HPLC-MS: *t*_R_ = 5.62 (10 min gradient of 60 to 95% of A in B). ^1^H-NMR: (300 MHz, CDCl_3_) δ (ppm): 3.81, 3.85, 3.86 and 3.89 (s, 3H, OCH_3_), 5.85 (s, 1H, H_4_), 6.45 (d, *J* = 2.5 Hz, 1H, 3-H), 6.52 (dd, *J* = 8.5 and 2.5 Hz, 1H, 5-H), 6.64 (d, *J* = 16.0 Hz, 1H, H_2_), 6.67 (d, *J* = 16.0 Hz, 1H, H_6_), 6.85 (d, *J* = 9.0 Hz, 1H, 6′-H), 6.90 (dd, *J* = 9.0 and 2.9 Hz, 1H, 5′-H), 7.08 (d, *J* = 2.9 Hz, 1H, 3′-H), 7.49 (d, *J* = 8.5 Hz, 1H, 6-H), 7.92 (d, *J* = 16.0 Hz, 1H, H_1_), 7.93 (d, *J* = 16.0 Hz, 1H, H_7_), 16.08 (br s, 1H, OH). ^13^C-NMR: (125 MHz, CDCl_3_) δ (ppm): 55.6 (2OCH3), 55.9 (OCH3), 56.2 (OCH3), 98.5 (C3), 101.5 (C_4_), 105.6 (C5), 112.6 (C4′), 113.1 (C6′), 116.8 (C3′), 117.3 (C1), 122.5 (C_2_), 124.9 (C1′), 125.1 (C_6_), 130.3 (C6), 135.0 (C_1_), 136.2 (C_7_), 153.0, 153.7, 160.1 and 162.8 (C2′, C5′, C2 and C4), 182.5 (C_3_-OH), 185.2 (CO). MS (ESI+): *m*/*z* 397 [M + H] ^+^. HRMS (ESI+): calc for C_23_H_24_O_6_ [M ]^+^ 396.1573, found 396.1580.

**(1E, 4Z, 6E) -1-(2,4-Dimethoxyphenyl)-7-(2-fluoro-4-methoxyphenyl)-5-hydroxyhepta-1,4,6 -trien-3-one (22).** Orange prisms (27%). From **20** and aldehyde **22**, following the same method described for the preparation of compound **23**. m.p.: 128–131 °C (MeOH). HPLC-MS: *t*_R_ = 3.28 (10 min gradient of 70 to 95% of A in B). ^1^H-NMR: (300 MHz, CDCl_3_) δ (ppm): 3.83 (s, 3H, OCH_3_), 3.85 (s, 3H, OCH_3_), 3.89 (s, 3H, OCH_3_), 5.81 (s, 1H, H_4_), 6.45 (d, *J* = 2.4 Hz, 1H, 3-H), 6.52 (dd, *J* = 8.5 and 2.4 Hz, 1H, 5-H), 6.60 (d, *J* = 16.0 Hz, 2H, H_2_), 6.63 (d, *J* = 16.0 Hz, 2H, H_6_), 6.65 (dd, *J* = 12.8 and 2.5 Hz, 1H, 3′-H)], 6.72 (dd, *J* = 8.7 and 2.5 Hz, 1H, 5′-H), 7.44 (dd, *J* = 9.0, 8.9 Hz, 1H, 6′-H), 7.50 (d, *J* = 8.9 Hz, 1H, 6-H], 7.68 (d, *J* = 16.0 Hz, 1H, H_1_), 7.91 (d, *J* = 16.0 Hz, 1H, H_7_). ^13^C-NMR: (125 MHz, CDCl_3_) δ (ppm): 55.6 (2OCH_3_), 55.8 (OCH_3_), 98.5 (C3), 101.5 (C_4_), 102.0 (d, *J* = 25 Hz, C3′), 105.6 (C5), 110.9 (d, *J* = 3 Hz, C5′), 116.0 (d, *J* = 12 Hz, C1′), 117.3 (C1), 122.4 (C_2_), 124.3 (d, *J* = 7 Hz, C_6_), 130.1 (d, *J* = 5 Hz, C6′), 130.4 (C6), 132.6 and 136.3 (C_1_, C_7_), 160.1, 162.9 (C2, C4), 162.3 (d, *J* = 11 Hz, C4′), 162.6 (d, *J* = 253 Hz, C2′), 182.3 (C_3_-OH), 185.1 (CO). MS (ESI+): *m*/I*z*: 385 [M + H]^+^. HRMS (ESI+): calc for C_22_H_21_FO_5_ [M ]^+^ 384.1373, found 384.1367.

General Procedure for the Synthesis of Tetrahydrocurcuminoids 23 and 24

The corresponding curcumin analogue (**17** or **21,** 0.25 mmol) was dissolved in 70 mL of a mixture of MeOH:THF (1:1) and Ph_2_S (0.0025 mmoL), and 10% Pd/C (0.025 mmoL) was added. The mixture was hydrogenated at 20 psi and room temperature overnight. The suspension was filtered and the solvent removed under vacuum. The crude product was purified by flash chromatography (gradient: 0 to 17% of EtOAc in hexane).

**(Z)-1,7-Bis(2,4-dimethoxyphenyl)-5-hydroxyhept-4-en-3-one (23).** From **17**. Pale oil. (85% yield). HPLC-MS: *t*_R_ = 5.25, tautomeric ratio: 72.1 (10 min gradient of 60 to 95% of A in B). ^1^H-NMR: (300 MHz, CDCl_3_) δ (ppm): 2.49–2.54 (m, 3H), 2.77–2.86 (m, 5H), 3.79 (s, 12H, OCH_3_), 5.45 (s, 1H, 4-H), 6.38–6.44 (m, 4H, 5, 5′, 3, 3′-H), 7.01 (d, *J* = 8,0 Hz, 2H, 6, 6′-H), 15.50 (br s, 1H, OH).^13^C-NMR: (75 MHz, CDCl_3_) δ (ppm): 24.3, 26.2 (C_1_, C_7_), 38.8, 44.0 (C_2_, C_6_)), 55.3, 55.5 (OCH_3_), 98.6 (C3, C3′), 99.4 (C_4_), 103.9 (C5, C5′), 121.6 (C1, C1′), 130.2(C6, C6′), 158.4, 159.6 (CCH_3_), 194.0 (C_3_-OH), 204.2 (CO)). MS (ESI+): *m*/I*z* 401 [M + H]^+^. HRMS (ESI+): calc for C_23_H_28_O_6_ [M]^+^ 400.1886, found 400.1870.

**(Z)-1-(2,4-Dimethoxyphenyl)-7-(2,5-dimethoxyphenyl)-5-hydroxyhept-4-en-3-one (24).** From **21**. Pale oil. (70% yield). HPLC-MS: *t*_R_ = 4.98 (10 min gradient of 60 to 95% of A in B). ^1^H-NMR: (300 MHz, CDCl_3_) δ (ppm): 2.49–2.58 (m, 3H), 2.74–2.90 (m, 5H), 3.75 and 3.78 (s, 3H, OCH_3_), 3.79 (s, 6H, OCH_3_), 5.46 (s, 1H, H_4_), 6.38–6.44 (m, 2H, 3-H, 5′-H), 6.70–6.78 (m, 3H, 3′, 4′, 6′-H), 7.01 (d, *J* = 8.1, 1H, 6-H), 15.49 (s, 1H, OH). ^13^C-NMR: (75 MHz, CDCl_3_) δ (ppm): 24.6 , 26.5 (C_1_, C_7_), 38.6, 44.1 (C_2_, C_6_,), 55.3, 55.5, 55.8 and 55.9 (OCH3), 98.6 (C3), 99.4 (C_4_), 103.9 (C5), 111.3 (C4′), 111.6 (C3′) 116.4 (C6′), 121.6 (C1), 130.2 (C6′), 130.4 (C1′), 151.8, 153.6, 158.4, 159.6 (COCH_3_), 193.8 (C_3_-OH), 204.0 (CO). MS (ESI+): *m*/I*z* 401 [M + H]^+^. HRMS (ESI+): calc for C_23_H_28_O_6_ [M]^+^ 400.1886, found 400.1881.

Synthesis of Compound 25

**(Z)-1,7-Bis(2,4-dihydroxyphenyl)-5-hydroxyhept-4-en-3-one (25).** To a solution of **23** in dry CH_2_Cl_2_ (0.66 mmol/15 mL), a 1M solution of Br_3_B in CH_2_Cl_2_ (9 equiv, 1.5 equiv for every O and/or N present in the molecule) was slowly added under Ar atmosphere. The reaction mixture was stirred at room temperature, and the progress of the reaction was monitored by thin-layer chromatography (TLC). After completion, the reaction was quenched by adding water (10 mL) and the precipitated solid filtered and washed with water. The product was extracted with EtOAc from the aqueous reaction mixture. The organic layer was washed with water until neutral pH, and then with brine and dried over anhydrous MgSO_4_. After removal of the solvent in vacuum, the crude product was purified by flash column chromatography eluting with CH_2_Cl_2_:MeOH, 20:1 solution. Yellow oil. 24% yield. HPLC-MS: *t*_R_ = 7.46 (10 min gradient, 30–95% of A in B). ^1^H-NMR: (400 MHz, CD_3_)_2_SO) δ (ppm): Keto-enol tautomeric equilibrium, 1:1, 2.45–2.68 (m, 8H), 3.66 (s, 2H, 4-CH_2_, keto form), 5.68 (s, 1H, 4-CH, enol form), 6.09–6.13 (m, 2H, 6, 6′-H), 6.23–6.26 (m, 2H, 3, 3′-H), 6.7–6.8 (m, 2H, 5, 5′-H), 8.95 (s, 1H, OH), 8.97 (s, 1H, OH), 9.13 (s, 1H, OH), 9.18 (s, 1H, OH), 15.59 (s, 1H, 4-OH). ^13^C-NMR: (100 MHz, CD_3_)_2_SO)) δ (ppm): 23.28, 23.65, 24.99, 25.34 (CH_2_, enol tautomer), 34.45, 38.10, 43.33 and 43.45 (CH_2_, keto tautomer), 56.19 (C_4_, keto tautomer), 99.21 (C_4_, enol tautomer), 102.35, 102.44 (C3, C3′), 105.82, 105.89 (C5, C5′), 117.22, 117.49 (C1, C1′), 129.87, 129.95 (C6, C6′), 155.71, 155.76, 155.84, 156.41, 156.45, 156.52, 156.56 (C-OH), 205.12 (C_3_-OH), 208.37 (CO). MS (ESI+): *m*/I*z m*/I*z*: 345 [M + H]^+^. HRMS (ESI-): calc for C_19_H_20_O_6_ [M]^+^ 344.1260, found 344.1260.

### 3.2. Biological Evaluation

#### Oocyte Expression and Electrophysiological Studies

All human nAChR cDNAs were cloned in derivatives of the pSP64T vector containing part of the pBluescript polylinker. Capped mRNA was synthesized in vitro using SP6 RNA polymerase, the mMESSAGEmMACHINE kit from Ambion (Thermo Fisher Scientific, Madrid, Spain), and the pSP64T derivatives mentioned above. Defoliculated Xenopus laevis oocytes were injected with 5 ng of each subunit cRNA in 50 nL of sterile water. All experiments were performed within 2–3 days after cRNA injection. Two-electrode voltage-clamp recordings were carried out in oocytes located in a chamber and perfused with a modified frog Ringer containing (in mM) NaCl 82.5, KCl 2.5, BaCl_2_ 2.5, MgCl_2_ 1, and HEPES 5 (pH 7.4). Barium, instead of calcium, was used as the extracellular divalent cation to avoid the presence of calcium-activated chloride currents. Unless otherwise specified, compounds were pre-applied in the bath for 2 min and then co-applied with ACh through a pipette held very close to the oocyte for fast application (18–22 mL/min), as previously described [67]. All experiments were performed at 22 °C. The holding potential was usually −80 mV. Current records were measured with Clampfit 10.0 (MDS Analytical Technologies, Sunnyvale, CA, USA). Normalized peak currents were obtained by dividing the maximum value of the current obtained in the presence of compound by the maximum value of the current obtained in control conditions (200 μM ACh, a value close to its EC_50_). Dose–response curves for the peak current obtained with ACh were fitted to the Hill equation: normalized current = Imax/(1 + (EC_50_/[ACh])^nH^). Data are expressed as mean ± SEM. All chemicals were purchased from Sigma-Aldrich (St. Louis, MO, USA).

## 4. Conclusions

With the aim of identifying new positive allosteric modulators of α7 nAChRs, we describe here the biological evaluation of a small collection of naturally occurring compounds, together with the synthesis and evaluation of new curcuminoids and tetrahydrocurcuminoids concerning their capacity as modulators of the α7 nAChRs with the aim of identifying new positive allosteric modulators.

The selected natural products include several polyhydroxyflavonoids, a polyhydroxy diphenylpropanone, phloretin, curcumin, and two curcuminoids, DMC and BDMC. The results obtained revealed the ability of phloretin, curcumin, and curcuminoids to enhance the ACh-induced currents in α7 nAChRs. Concerning phloretin (**11**), a polyhydroxydiphenylpropanone, its tendency to increase the current is in agreement with our previously described findings for this chemical series [19]. On the other hand, regarding curcumin, our results confirm its capacity as PAM of the α7 receptors, which together with the even better values found for curcuminoids DMC and BDMC prompted us to the preparation of new curcumin analogues. Three of the new compounds prepared, the three tetrahydrocurcuminoids, responding to a bis(2,4-diphenyl) symmetric substitution pattern (**17**, **23** and **25**), were able to activate the α7 nicotinic receptors in an allosteric manner. The best result was found for the tetrahydrocurcumin analogue **23**, a tetramethoxy substituted compound that increased the current at superior levels than its tetrahydroxy analogue **25**. This is in contrast with the behavior previously observed for our chalcone and diphenylpropanone series, for which only the polyhydroxy derivatives behave as PAMs of the α7 nAChRs.

These results confirm the interest of curcumin and curcuminoids in terms of activation of α7 nAChRs and additionally revealed the potential of the tetrahydrocurcumin as a new scaffold to be able to positively modulate these receptors. The results are very promising and open the way to further research in order to fully establish these new scaffolds as a new series of α7 nAChRs PAMs.

## Figures and Tables

**Table 1:** Structure of commercial naturally occurring compounds **1** to **14** and their potentiation percentage values of α7 nAChR currents.

Compound Number	Molecular Structure	% ± s.e. Current vs. ACh [200 µM] ^a^
**1** **Apigenin**	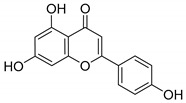	108 ± 4
**2** **Quercetin**	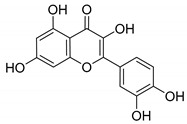	130 ± 10
**3** **Luteolin**	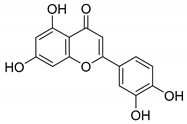	125 ± 5.5
**4** **Genistein**	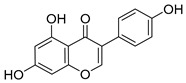	154 ± 10
**5** **Gossypetin**	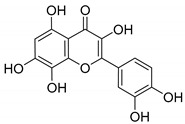	115 ± 2
**6** **Kaempferol**	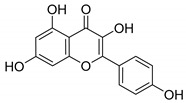	155 ± 5
**7** **Myricetin**	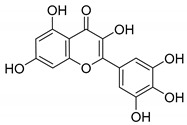	97 ± 6
**8** **Eriodictyol**	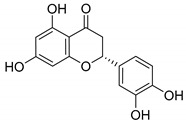	108 ± 2.5
**9** **Naringenin**	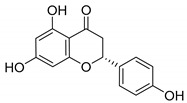	105 ± 3
**10** **Taxifolin**	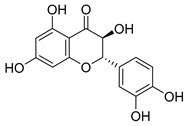	107 ± 5.5
**11** **Phloretin**	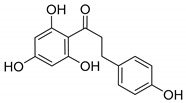	231 ± 0.5
**12** **Curcumin**	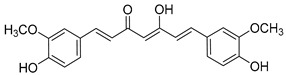	148 ± 43
**13** **Demethoxycurcumin (DMC)**	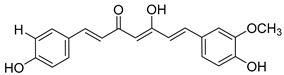	408 ± 18
**14** **Bisdemethoxycurcumin (BDMC)**	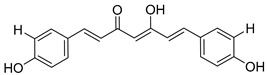	469 ± 42

^a^ Compounds were tested at a unique dose of 10 µM, which was co-applied with 200 μM ACh. Responses were recorded at −80 mV and normalized with respect to that shown by only ACh (200 μM). Data are taken from 6–10 oocytes from at least 2 donors.

**Table 2:** Effects of compounds **17** and **21–25** on α7 nAChR currents.

Compound Number	Molecular Structure	% ± s.e. Current Vs. ACh [200 µM] ^a^
**17**	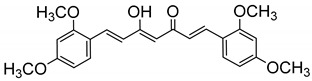	143 ± 3.5
**21**	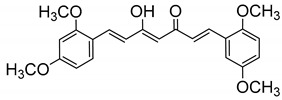	107 ± 4
**22**	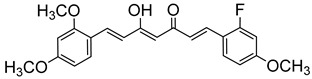	153 ± 17
**23**	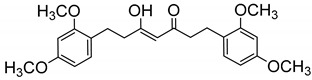	498 ± 7
**24**	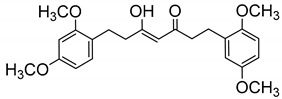	53.5 ± 3.5
**25**	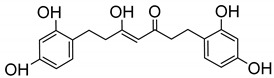	251 ± 24

^a^ Compounds were tested at a unique dose of 10 µM**,** which was co-applied with 200 μM ACh. Responses were recorded at −80 mV and normalized with respect to that shown by only ACh (200 μM). Data are taken from 6–10 oocytes from at least 2 donors.

## Data Availability

Data is contained within the article.
